# Commentary: Artificial Intelligence and Statistics: Just the Old Wine in New Wineskins?

**DOI:** 10.3389/fdgth.2022.923944

**Published:** 2022-05-20

**Authors:** Anne A. H. de Hond, Ben van Calster, Ewout W. Steyerberg

**Affiliations:** ^1^Clinical Artificial Intelligence Implementation and Research Lab, Leiden University Medical Centre, Leiden, Netherlands; ^2^Department of Medicine (Biomedical Informatics), Stanford University, Stanford, CA, United States; ^3^Department of Biomedical Data Sciences, Leiden University Medical Centre, Leiden, Netherlands; ^4^Department of Development and Regeneration, KU Leuven, Leuven, Belgium

**Keywords:** artificial intelligence, machine learning, statistics, methodology, discrimination

We write to expand on Faes's et al. recent publication “*Artificial intelligence and statistics: Just the old wine in new wineskins?*” (1). The authors rightly address a lack of consensus regarding terminology between the statistics and machine learning fields. Guidance is needed to provide a more unified way of reporting and comparing study results between the different fields, as far as these can be separated.

Prediction models can be based on traditional statistical learning methods, such as regression, and modern machine learning approaches, such as tree-based methods (random forests, XGBoost) and neural networks. These models can be evaluated along several evaluation axes. Measures for discrimination typically quantify the separation between low vs. high-risk subjects, independent of the event rate (2). Classification is often grouped under discrimination, but classification measures are dependent on the decision threshold used to define subjects as high-risk vs. low risk. Moreover, classification performance is affected by calibration, which relates to the reliability of the estimated risks (3). Overall performance measures are also available, including Brier score and measures for explained variability (*R*^2^), which reflect both discrimination and calibration performance. Lastly, measures for clinical utility have been proposed, which consider the clinical context with respect to the event rate and the decision threshold to define high vs. low risk (4, 5). Major differences can be observed in the measures commonly used across these axes to evaluate predictive performance in the statistics and machine learning fields.

We here highlight key measures focusing on discriminative ability and clinical utility [or effectiveness (6)]. Table 1 provides a non-exhaustive overview. All measures relate to the evaluation of probability predictions for binary outcomes. They are derived from the 2 × 2 confusion matrix for specific or consecutive decision thresholds. We reflect on these measures below:

**Table 1:** Evaluation measures from statistics and machine learning fields.

**Evaluation measures**	**Field (statistics/machine learning)**	**Definition**
**Discrimination measures (decision threshold independent)**		
Area under the receiver operating characteristic-curve (AUROC)	S/ML	The receiver operating characteristic (ROC) curve plots sensitivity as a function of 1-specificity. The baseline is fixed. The area under the ROC-curve can be compared across settings with different event rates
Area under the precision recall-curve (AUPRC)	ML	The precision recall curve plots the precision (positive predictive value) as a function of sensitivity. The baseline is determined by the ratio of positive predictions and total predictions. The area under the precision recall curve cannot be compared across settings with different event rates and ignores true negatives
**Classification measures (decision threshold dependent)**		
Crude accuracy	ML	Crude accuracy is the number of true positive and negative predictions divided by the total number of cases
Sensitivity (recall)	S/ML	The sensitivity is the number of true positive predictions divided by the number of true positive cases at a specified probability threshold
Specificity	S/ML	The specificity is the number of true negative predictions divided by the number of true negative cases at a specified probability threshold
Positive predictive value (precision)	S/ML	The positive predictive value (PPV) is the number of true positive predictions divided by the total number of positive predictions at a specified probability threshold
Negative predictive value	S/ML	The negative predictive value (NPV) is the number of true negative predictions divided by the total number of negative predictions at a specified probability threshold
*F*_β_-score	ML	The *F*_β_-score is the harmonic mean of sensitivity and positive predictive value controlled by the β coefficient: $F_{\beta }=\left(1+\beta^{2}\right)*\frac{PPV*sensitivity}{\beta^{2}*PPV+sensitivity}$. When false positives are more important than false negatives, the β coefficient is set to be smaller than 1. When false negatives are more important than false positives, the β coefficient is set to be larger than 1. Popular installments of the *F*_β_-score are the *F*_1_- and *F*_2_-score. The *F*_1_score implies equal weight for false negatives and false positive classifications, which is “absurd” for most medical contexts (7)
**Measures related to clinical utility**		
Net Benefit	S	Net Benefit is a weighted sum of true positive (TP) and false positive (FP) predictions at a given decision threshold (t): $NB=\left(TP-\frac{t}{1-t}*FP\right)/N$. Net Benefit can be plotted over a range of decision thresholds resulting in a decision curve (4)
Relative utility	S	Relative utility is the maximum net benefit of risk prediction at a given decision threshold divided by the maximum net benefit of perfect prediction. A relative utility curve plots relative utility over a range of decision thresholds (8)

The precision recall-curve and F1-score are often described in the machine learning field as “superior for imbalanced data” (9, 10). Indeed, recall (sensitivity) and precision (positive predictive value) are evenly weighted in the computation of the area under the precision recall-curve (AUPRC) and the F1-score. However, imbalanced data is usually not considered problematic for classic statistical learning (such as logistic regression), except for edge cases where the event rate is exceptionally low. Because the precision recall-curve and F1-score are event rate dependent, we cannot directly compare model performance for settings with a different event rate. Also, the precision recall-curve ignores true negatives and therefore is not a measure of discrimination according to the above definition. In contrast, the classic area under the receiver operating characteristic curve (AUROC) is event rate independent, which is a hall mark of a discrimination measure (2). Similarly, sensitivity (fraction true positive) and specificity (fraction true negative) can, at least in theory, be considered as independent of event rate.

Some measures are considered outdated in the classic statistical learning field, while still popular in the machine learning field. Such a measure is the crude accuracy (the fraction of correct classifications). Crude accuracy is event rate dependent, e.g., a 99% accuracy is the minimum for a setting with 1% event rate and classifying all subjects as “low risk.”

Decision analytical approaches move away from pure discrimination and toward clinical utility. Net benefit is the most popular among some recently proposed measures for clinical utility (4, 5). It is derived from a decision analytical framework and weighs sensitivity and specificity by clinical consequences. Net benefit has a clear interpretation when compared to treat-all and treat-none strategies (4, 5).

In conclusion, measures that are affected by the event rate are common in the machine learning field, such as the AUPRC, F1-score, and crude accuracy. They impede the comparison of model performance across different settings. The medical decision-making context is better captured in modern measures such as Net Benefit, which not only consider the event rate but also the clinical consequences of false-positive vs. true-positive decisions (harm vs. benefit), rather than arbitrary weighting these costs (7). We recommend that the aim of the evaluation of a model should determine our focus at clinical performance (discrimination, calibration), or clinical utility, with quantification by appropriate measures.

## Author Contributions

AH, BC, and ES conceived the idea, wrote the initial draft, edited, and approved the final manuscript. All authors contributed to the article and approved the submitted version.

## Conflict of Interest

The authors declare that the research was conducted in the absence of any commercial or financial relationships that could be construed as a potential conflict of interest.

## Publisher's Note

All claims expressed in this article are solely those of the authors and do not necessarily represent those of their affiliated organizations, or those of the publisher, the editors and the reviewers. Any product that may be evaluated in this article, or claim that may be made by its manufacturer, is not guaranteed or endorsed by the publisher.
